# Interleukins IL33/ST2 and IL1-β in Intrauterine Growth Restriction and Seropositivity of Anti-*Toxoplasma gondii* Antibodies

**DOI:** 10.3390/microorganisms12071420

**Published:** 2024-07-12

**Authors:** Karen Franco-De León, Eva Elizabeth Camarena, Ana Laura Pereira-Suárez, Ernesto Barrios-Prieto, Andrea Soto-Venegas, Zamira Helena Hernández-Nazara, Yithzel Guadalupe Luna Rojas, María de la Luz Galván-Ramírez

**Affiliations:** 1 Departamento de Microbiología y Patología, Centro Universitario de Ciencias de la Salud, Universidad de Guadalajara, Guadalajara 44340, Jalisco, Mexico; 2 Departamento de Ginecología y Obstetricia, Hospital Civil Juan I. Menchaca, Guadalajara 44340, Jalisco, Mexico; 3 Unidad de Medicina Materno Fetal, Hospital Civil Juan I. Menchaca, Guadalajara 44340, Jalisco, Mexico; 4 Instituto de Investigación en Enfermedades Crónico Degenerativas, Centro Universitario de Ciencias de la Salud, Universidad de Guadalajara, Guadalajara 44340, Jalisco, Mexico

**Keywords:** toxoplasmosis, intrauterine growth restriction, IL-33, IL-1β

## Abstract

*Toxoplasma gondii* (*T. gondii*) is the causal agent of toxoplasmosis. It may produce severe damage in immunocompromised individuals, as well as congenital infection and intrauterine growth restriction (IUGR). Previous reports have associated interleukin IL-33 with miscarriage, fetal damage, and premature delivery due to infections with various microorganisms. However, IL-33 has not been associated with congenital toxoplasmosis. The sST2 receptor has been reported in patients who have had recurrent miscarriages. On the other hand, IL-1β was not found in acute *Toxoplasma* infection. Our aim was to analyze the associations between the serum levels of IL-33 and IL-1β in IUGR and toxoplasmosis during pregnancy. Eighty-four serum samples from pregnant women who had undergone 26 weeks of gestation were grouped as follows: with anti-*Toxoplasma* antibodies, without anti-*Toxoplasma* antibodies, IUGR, and the control group. IgG and IgM anti-*T. gondii* antibodies, as well as IL-33, ST2, and IL-1β, were determined using an ELISA assay. Statistical analyses were performed using the Pearson and Chi-square correlation coefficients, as well as the risk factors and Odds Ratios (ORs), with a confidence interval of 95% (CI 95). The results showed that 15/84 (17.8%) of cases were positive for IgG anti-*Toxoplasma* antibodies and 2/84 (2.38%) of cases were positive for IgM. A statistically significant difference was found between IUGR and IL-33 (*p* < 0.001), as well as between ST2 and IUGR (*p* < 0.001). In conclusion, IUGR was significantly associated with IL-33 and ST2 positivity based on the overall IUGR grade. No significant association was found between IUGR and the presence of anti-*Toxoplasma* antibodies. There was no association between IL-1β and IUGR. More research is needed to strengthen the utility of IL-33 and ST2 as biomarkers of IUGR.

## 1. Introduction

The worldwide incidence of congenital *Toxoplasma gondii* (*T. gondii*) infection has reached 190,100 cases per year, with an incidence rate of 1.5 cases of congenital toxoplasmosis per 1000 births. America has presented the highest infection prevalence [1].

*T. gondii* infection is considered to be in the TORCH (toxoplasmosis, Rubella, Cytomegalovirus, and Herpes simplex virus) group that is transmitted through the placenta during the gestation process. Such infections can cause significant short- and long-term damage, particularly in premature infants. Infection with *T. gondii* can result in miscarriage [2,3]. The difference in the clinical pictures of damage from *T. gondii* infection are dependent on the trimester of pregnancy in which the mother acquired the infection. Moreover, premature labor and intrauterine growth restriction (IUGR) could occur at this stage, which is a stage that is close to the end of gestation [3,4]. The first trimester, which can involve neurological syndromes such as hydrocephalus, microcephaly, microphthalmia, intracerebral calcifications, and retinochoroiditis, as well as hepatomegaly, splenomegaly, and hyperbilirubinemia, is sometimes accompanied by thrombocytopenia [3,4]. In the second trimester, cerebral calcifications, hepatomegaly, anemia, epilepsy, and retinochoroiditis can appear. During the third trimester, most cases of infection may be develop in asymptomatic newborns, presenting retinochoroiditis, blindness, strabismus, epilepsy, hearing disorders, or neurological deficits months or years later in childhood (and this can even occur in early adulthood in addition to the fact that premature labor could occur at this stage as it is close to the end of the gestation and intrauterine growth restriction (IUGR) [3,4]).

IL-33 is a pro-inflammatory interleukin and belongs to the IL-1α, IL-18, IL-33, IL-1RA, and IL-1β families. This interleukin plays a key role in the Th1 immune response. IL-1α and IL-1β share the IL-1RAcP (IL-1 receptor accessory protein) co-receptor for IL-33 [5,6,7], and they also share the caspase-1 enzyme with IL-1 and IL-18. The caspase-1 enzyme is responsible for synthesizing these interleukins as precursor molecules (NALP- 3). It is activated due to existing tissue damage, infection, or mechanical stress [6,7,8,9]. However, other authors have proposed that IL-33 may be active independently of caspase-1 [10]. They have demonstrated that caspases 3 and 7 process IL-33, bind its (receptor suppression of tumorigenicity) ST2, and do not depend on caspase processing [10,11].

The IL-33 in endothelial cells triggers the activation of the Th2 response, specifically, the induction of the interleukins IL-4, IL-5, and IL-13 [11], as well as the activation of group 2 innate lymphoid cells (ILCs) [12]. IL-33 has been associated with miscarriage, preterm labor, and preeclampsia, the last of which has been associated with IL-33 and ST2 during the sixth week of gestation [13]. On the other hand, the pro-inflammatory response is antagonistic to tolerogenic cytokines, which are necessary for the successful termination of pregnancy. This pro-inflammatory response can trigger abortions [14,15].

In a study conducted on 300 Iranian women who were at risk of recurrent pregnancy loss and a control group comprising 300 healthy women pregnancies with successful delivery, an analysis of polymorphisms and the interleukins IL-10, IL-18, and IL-33 found that higher frequencies of interleukin IL-33 are associated with the risk of suffering miscarriage [13]. Additionally, the serum IL-33 levels in pregnant women who had miscarried were much higher than those reported in normal pregnancies [13,15]. A study of Brazilian pregnant women found higher levels of IL-33, which were also associated with the IgG antibodies anti-*T. gondii*. Furthermore, pregnant and multiparous women were more found to likely to be infected compared to primiparous women [16].

The receptor of IL-33 is ST2, which is also known as IL-1RL1, IL-1R4, T1, and IL-33R. ST2 has two isoforms: a complete transmembrane form (ST2L) and a soluble form (sST2). It is expressed in mast cells, macrophages, NK cells, and Th2 lymphocytes [11]. To initiate the signaling pathway, IL-33 must bind ST2 and allow interaction with the IL-1 receptor accessory protein (IL-1RAcP), which acts as a co-receptor forming a dimer with ST2. This binding recruits the myeloid differentiation primary response protein 88 (MYD88), as well as the IL-1R-associated kinase 1 (IRAK1) and IRAK4; the TNF factor 6-associated receptor (TRAF 6); and it activates the nuclear factor κB (NF-κB) and mitogen-activated kinases, such as extracellular signal-regulated kinases (ERKs), mitogen-activated protein kinases (p38), and c-terminal kinase N-terminal kinases Jun (JNK) inducing immune response Th2. If sST2 binds IL-33 directly, it will act as a decoy receptor to compete with ST2L, preventing binding to IL-1RAcP and the Th2 response, thus leaving the Th1 response [12].

The sST2 receptor has been reported in patients who have had recurrent miscarriages, and it exhibits a correlation with decidual macrophages and an M1 profile that favors the elimination of damaged cells using efferocytosis [15].

IL-1β is part of the IL-1 family. Various immune cells are involved in the synthesis of IL-1β, with macrophages being the most responsible, although monocytes, dendritic cells, fibroblasts, endothelial cells, keratinocytes, and hepatocytes are also present [8,12]. IL-1β is a monomer of 153 amino acids, triggering fever, the activation of T lymphocytes (particularly in Th17 differentiation), and the activation of macrophages [11]. For the mature form of IL-1β to occur, the involvement of caspase-1 is necessary [17].

The receptor of IL-1β is IL-1R1. IL-1β also shares the accessory protein IL-1RAcP (which is also known as IL-1R3) and NALP3 (for the processing of monocytes) with IL-33 [6].

In negative signaling, IL1-β will have the receptor antagonist IL-1R2, which, unlike IL-1R1, does not have a transmembrane structure, making the Toll domain of the IL-1 receptor (TIR) necessary. The IL-1R2 receptor has soluble forms that will also inhibit IL-1β signal transduction.

A study demonstrated that components of the NLRP7 inflammasome and IL-1β circulating levels were increased in IUGR [18]. Likewise, growth-restricted preterm newborns showed elevated concentrations of IL-1β on day 14 post-natal [19]. On the other hand, in the colostrum of women at the extremes of reproductive age, IL-1β and IL-6 presented higher levels compared with those in a group of women between 20 and 24 years, which reflects a better colostrum composition that is influenced by maternal age with respect to maturity in the biological sense [20].

The immune response to *T. gondii* infection depends on the genetic diversity of the parasite and the host’s immune status. For an efficient immune response, the intervention of innate immunity is necessary; when coupled with an effective adaptive response, this will activate a cytotoxic mechanism, regulating infection and the consolidation of immunological memory in the face of parasites [21].

At the beginning of the immune response, in innate immunity, Toll-like receptors (TLRs) intervene as they are capable of recognizing the Pathogen-Associated Molecular Patterns (PAMPs) of *T. gondii*. These participate in the activation of myeloid differentiation factor 88 (MYD88), which is necessary for the activation of the interleukin 12 (IL-12) and T helper 1 (Th1) cells, and they are part of the signaling system of CD4+ cells in addition to the production of INF-γ. The CD4+ cell complex is responsible for notifying the B cells of an antigen through cytokines using the major histocompatibility complex II (MHCII); as a consequence, the B cells will recognize and bind the antigen. In the immune response to *T. gondii* infection, the participating TLRs are TLR2, TLR4, and TLR5 [4,22].

INF-γ, through antimicrobial and oxidative metabolic mechanisms, seeks to eliminate the presence of *T. gondii* in the host due to suppression of parasite cell growth and starvation by deficiency tryptophan, as well as by inducing indoleamine 2,3-dioxygenase, nitric oxide-mediated blockade of mitosis, and arginine deficiency, which are necessary in the replication of parasites [23].

In the adaptive response, IL-12 induces Th1 cells, which promote the presence of cytotoxic CD8+ T cells, to control the number of tissue cysts, and they are also vital for the regulation of *Toxoplasma gondii* infection. The presence of macrophages, NK (natural killer), T lymphocytes, and cytokines such as IL-12, IL-1β, IFN-γ, and TNF-α constitute elements for resistance to the parasite. IL1-β acts on the Th1, Th17 lymphocytes, and innate lymphoid cells in inflammatory processes and on the development of Th1 immunity [17].

However, for the resistance at an early stage of *T. gondii* infection, the intervention of CD4+ cell complex cytokines is necessary, such as IL-5, IL-4, IL-10, and IL-6. These anti-inflammatory cytokines activate the response to infection through MHC II with the proteins from the parasite for the processing of the antigen and the induction of the production of IgM antibodies that appear on the seventh-day post-infection, as well as in the IgG presenting two weeks after infection [21], thus triggering the production of IL-2 for the development of CD8+ cells. In addition, the actions of the group of Th2-type cytokines counteract those carried out by Th1-type cytokines. An example of this is the regulation of INF-γ by IL-10, where IL-10 blocks the INF-γ production of macrophages. The correct balance of the Th1 and Th2 response is critical for effective infection control [4,21].

**Figure 1:**
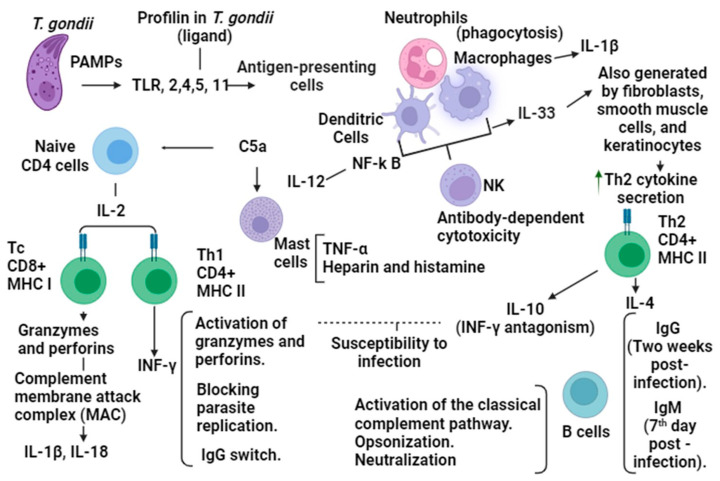
The immune response to *Toxoplasma gondii* and the possible intervention of IL-33 is shown. *Toxoplasma gondii* (*T. gondii*), Pathogen-Associated Molecular Patterns (PAMPs), Toll-like receptors (TLR), interleukin 12 (IL-12), interleukin 2 (IL-2), interleukin 1 beta (IL-1β), gamma interferon (IFNγ), immunoglobulin G (IgG), immunoglobulin M (IgM), helper T cells (Th), interleukin 10 (IL-10), interleukin 4 (IL-4), cytotoxic T lymphocytes (Tc), dendritic cells (DC), natural killer cells (NK), interleukin 33 (IL-33), major histocompatibility complex (MHC), cluster of differentiation 4 positive (CD4+), cluster of differentiation 8 positive (CD8+), and innate lymphoid cells (ILC 1).

The immune response for long-term resistance to *Toxoplasma gondii*, which is necessary to activate CD8+ T lymphocytes that recognize infected cells, initiates a specific response of MHC II that identifies foreign material. Leads of proteasome modifications are required for the activation of proteases. Memory generation is carried out by CD8+ T cells, which go through a process of contraction where some short-lived effector cells (SECs) survive apoptosis and become memory precursor effector cells (MPECs) [17] (Figure 1).

IUGR is considered when the estimated fetal weight is less than the 10th percentile for the gestational age. It is the intrinsic condition of compromise of placental support for the fetus, causing hypoxia and inappropriate nutrition, as well as a pathological restriction of the fetal genetic potential for growth.

Symmetrical IUGR (intrinsic cause) implies restriction of body and brain growth. Symmetrical IUGR comprises placental abnormalities, such as vascular abnormalities; abruptions; ruptures; congenital infections; chromosomal disorders; and factors related to the mother; as well as the use of teratogenic drugs or narcotics, alcohol consumption, and smoking [23,24,25]. In asymmetrical IUGR (extrinsic cause), brain growth is not affected. The most common causes are diabetes mellitus, kidney disease, and smoking. Toxoplasmosis is an infection that is involved in IUGR [23,24,25,26].

The purpose of this study was to determine the association of IL-33, ST2, IL-1β, and *T. gondii* infection in women with intrauterine growth restriction.

## 2. Materials and Methods

### 2.1. Patients

Pregnant women whose fetuses were diagnosed with intrauterine growth restriction (IUGR) were recruited from the Obstetric Gynecology Service and Maternal Fetal Medicine Unit of the New Civil Hospital of Guadalajara, Juan I. Menchaca in Guadalajara, Jalisco, Mexico, from March to August 2020–2022.

The sample size (N) was calculated with the following formula:

Z^2^ (p × q) N/(d)^2^ + Z^2^ (p × q),

where Z = 1.96 (for 95% confidence); d = 0.05 (precision); p = 0.22 (estimated proportion of “presence of congenital toxoplasmosis”); q = 0.78 (estimated proportion of “absence of congenital toxoplasmosis”); and N = 162 (the mean birth rate of the New Hospital Civil de Guadalajara, Juan I Menchaca).

### 2.2. Inclusion Criteria

This work is a cross sectional study. In total, 84 serum samples from pregnant women (who were past 26 weeks (third trimester) of gestation) were included. Based on the results of the ultrasonography, clinical backgrounds, and the presence of anti-*T. gondii* antibodies, the sera of the patients were divided into 3 groups: Group I, from pregnant women with intrauterine growth restriction (IUGR) and positive for anti-*T. gondii* antibodies *n =* 16; Group II, with IUGR and negative for anti-*T. gondii* antibodies *n =* 49; and Group III (control group), without IUGR and negative for anti-*T. gondii* antibodies *n* = 19.

### 2.3. Exclusion Criteria

Patients with diagnoses of membrane ruptures, autoimmune diseases, active or recent infections, TORCH *Rubella*, *Cytomegalovirus*, or *Herpes*, and those who presented other malformations (e.g., gastroschisis, holoprocencephalea, omphalocele, chromosomopathies) were excluded.

### 2.4. Diagnosis of IUGR

Fetal Doppler velocimetry duplex ultrasound of the umbilical artery was used [24]. The following classifications were used: Early IUGR was defined as before 32 weeks of gestation and late after 32 weeks at the estimated fetal weight, the percentiles thereof for the gestational age, and the Doppler value for the classification of IUGR. Type I: when the fetal weight is below the 10th percentile and has an abnormal Doppler value when the Doppler value should be normal and the weight is less than the 3rd percentile is the classification for IURG. Type II: when the estimated fetal weight is below the 10th percentile and the Doppler value shows an absent umbilical artery of the diastolic flow in more than 50% of the measured cycles (where two measurements have a separation of more than 12 h). Type III: when the fetal weight is less than the 10th percentile and the reverse umbilical artery has a diastolic flow in more than 50% of the measured cycles (with two measurements 6 to 12 h apart). Type IV: when the fetal weight is less than the 10th percentile with a non-tranquilizing fetal status and reverse diastolic flow in the ductus venous and pathological cardiotocography recording (where the variability is less than five beats per minute in the absence of any sedative medication) [23,24,25,26].

### 2.5. Serological Testing for T. gondii Antibodies

The blood samples were processed at the Institute for Research in Chronic Degenerative Diseases at the University Center of Health Sciences of the University of Guadalajara. The serum samples were obtained using centrifugation and kept frozen at −20 °C until needed for processing. The samples, standards, and controls were processed in duplicate. The IgG e IgM detection of Anti-*T. gondii* antibodies was achieved via ELISA (Platelia TM Toxo; Bio-Rad, IgG #72840 and IgM #72841, Marnes-la-Coquette, France). The microplates were recovered with inactivated *T. gondii* antigens. The plates were read at a 450/620 nm wavelength. The optical density values obtained were plotted along a standard curve to determine the levels of the antibody titers (IU/mL). The coefficient of variation for the intra-assays and inter-assays was 5.96% and 10.2%, respectively; the sensitivity of IgG was 98.3%; and the specificity was 100%. Regarding the IgM antibodies, only 2/84 samples (2.38%) were positive. Concerning IgM, the intra-assay and the inter-assay coefficient of variation were 5.96% and 10.1%, respectively. The sensitivity was 93% and the specificity was 99.9%.

### 2.6. IL-33 Assay

The serum concentrations of IL-33 were determined with the Human IL-33 ELISA Kit (RAB0297, Sigma Aldrich, St. Louis, MO, USA). Briefly, 100 µL of each standard and sample, previously diluted at a 1:2 ration, was added by duplicate and incubated overnight at 4 °C with gentle shaking. Then, the plate was washed with 300 μL of 1X Wash Solution, which was then decanted and dried on paper towels. Afterward, 100 μL of the detection antibodies was added to each well and incubated for 1 h at room temperature with gentle agitation and washing, as described above. After 100 µL of prepared streptavidin solution was added, the samples were incubated for 45 min at room temperature, and they were then gentle shaken and washed. Next, 100 µL of TMB substrate reagent was added to each well, covered to protect it from light, and then incubated for 30 min at room temperature in the dark with gentle agitation. Afterward, 50 μL of stop solution was added to each well and read at a wavelength of 450 nm. The serum concentrations were determined using a standard curve. The cutoff value was 2 pg/mL, and samples with values above this were considered positive. The intra-assay IL-33 coefficient of variation was 2.9% and the inter-assay was 8.7%. The sensitivity was 93.75% and the specificity was 94.10%.

### 2.7. Human ST2 Assay

The serum concentrations of ST2 were determined with a Human ST2 ELISA Kit (RAB0281, Sigma Aldrich, St. Louis, MO, USA). Briefly, 100 µL of each standard and sample, which were previously diluted at a ratio of 1:2, were added by duplicate and incubated overnight at 4 °C with gentle shaking. Afterward, the buffer was removed, and the plates were dried using paper towels, with washing repeated four times. Next, 100 µL of the detection antibody, prepared 1X, was added to each well, covered, and incubated for 1 h at room temperature with gentle shaking. Then, they were washed again, as described above. Afterward, 100 µL of prepared streptavidin solution was added to each well, incubated for 45 min at room temperature with gentle agitation, and then washed again. Later, 100 µL of TMB substrate was added and incubated for 30 min at room temperature. Then, 50 µL of stop solution was added to each well. Absorbance was determined at a 450 nm wavelength. The coefficient of variation was 3.9% for the intra-assay ST2, and inter-assay was 10.6%. The sensitivity was 45.1% and the specificity was 93.8%.

### 2.8. IL-1β/IL-1F2 Quantikine^®^ ELISA

The serum concentrations of IL-β were determined with the Human IL-1β/IL-1F2 *Quantikine* Kit (SLB50 R&D Systems, Minneapolis, MN, USA). Briefly, in this procedure, 50 μL of the RD1-83 assay diluent was added, plus 200 μL of the standard, control, or sample per well by duplicate, and the plate was incubated for two hours at room temperature. Subsequently, it was aspirated and washed four times with 400 μL of washing buffer, and then the plate was inverted on paper towels. Next, 200 µL of human IL-1β conjugate was added to each well and incubated for two hours at room temperature. The previously described wash was repeated, after which 200 μL of substrate solution was added to each well and incubated for 20 min at room temperature and protected from light. Then, 50 μL of stop solution was added to each well. The color of the wells changed to yellow. Finally, the optical density was read in a microplate reader at 450 or 570 nm. The coefficient of variation was 4.28% for Intra-Assay IL-1β, and the inter-assay was 11.2%. The sensibility was 25.42% and the specificity was 84.0%.

### 2.9. Statistical Analysis

The SPSS Version 20.0 and SPSS (v. 18) packages (IBM, Los Angeles, CA, USA) were used to perform all statistical analyses. The quantitative variables included age. The statistical significance between the two groups (positive vs. negative in ELISA) for the differences observed in these variables was obtained with 2 × 2 tables, which were also used to calculate the Pearson (r) and Spearman (Rho) correlation coefficients and Chi square test between the ELISA IgG values and clinical variables.

For all multiple-group comparisons, one-way ANOVA was performed, and a *p* < 0.05 was considered statistically significant throughout.

## 3. Results

### 3.1. Prevalence of Toxoplasma Infection

**Figure 2:**
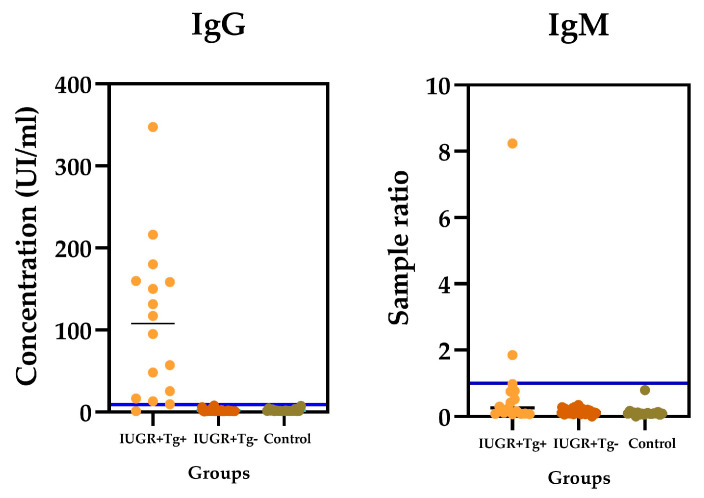
Anti-*T. gondii* antibody concentrations: the IgG and IgM antibodies of each group. Group I: positive IUGR and *Toxoplasma* antibodies (orange color), Group II: positive IUGR and negative for *T. gondii* antibodies (brown color); and Group III: negative for *T. gondii* and IUGR (green color), purple line is the cutoff value.

The mean age of the women studied was 24.11 years, with a standard deviation of +/− 8.3 years, a minimum limit of 14 years, and a maximum of 42 years. In the presence of anti-*T. gondii* antibodies, 2/84 (2.38%) samples were positive for IgM, and 15/84 sera were positive for IgG. The IgG anti-*Toxoplasma* antibodies reflected an overall prevalence of 15/84 samples (17.8%) (Figure 2).

### 3.2. Interleukins

**Figure 3:**
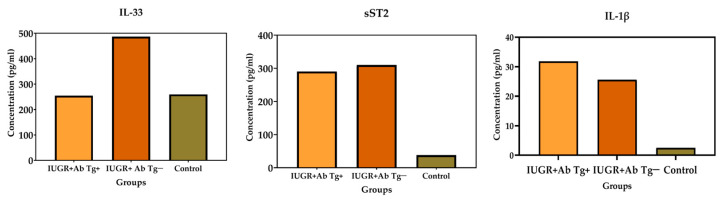
Concentrations of interleukins IL-33, IL-1β, and ST2 in the different groups. Group I: IUGR+ anti-*Toxoplasma* IgG antibodies positive (AbTg+) (orange color n = 16); Group II: IUGR+ anti-*Toxoplasma* antibodies negatives (AbTg−) (brown color n = 19); and Group III: AbTg−IUGR− (green color n = 49) Regarding IL-33. Increased values were observed predominantly in Group II (C).

The IL-33 was positive in 65/84 (77.38%) cases. The patients in the groups with and without anti-*Toxoplasma* antibodies and intrauterine growth restriction were positive, with higher concentration levels compared to IL1-β and ST2 (Figure 3).

**Figure 4:**
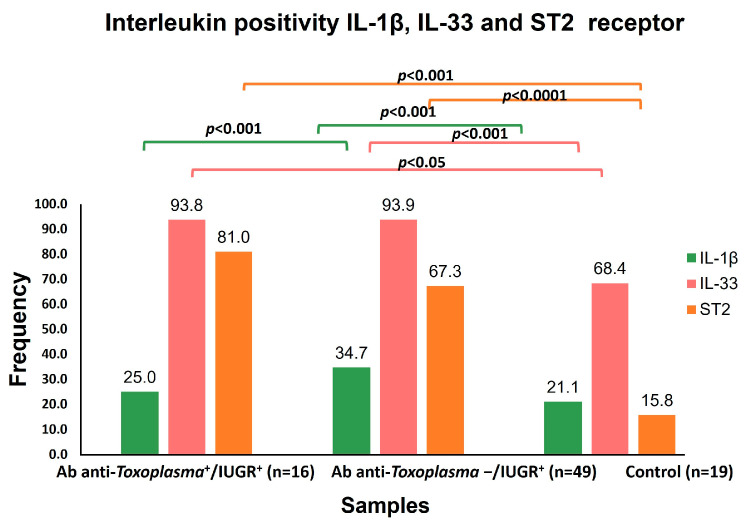
The IL-33, IL-1β, and ST2 concentrations among groups were compared using ANOVA. The IL-33 positivity values of the groups with IUGR were statistically significant (*p* < 0.05, *p* < 0.001). For IL-1β, a statistical significance of *p* < 0.001, *p* < 0.001 arose during the comparison of the positivity of Group I (Ab anti-Tg+/IUGR+). For ST2 a statistical significance of *p* < 0.001, *p* < 0.0001 with that of the control group.

The global response of IL-1β was low as only 6/84 (7.14%) cases were positive, and an increase in the concentration values was found in Group II, which is in contrast with Groups I and III. The mean IL-33, IL-1β, and ST2 concentrations were compared through ANOVA (Figure 4).

**Figure 5:**
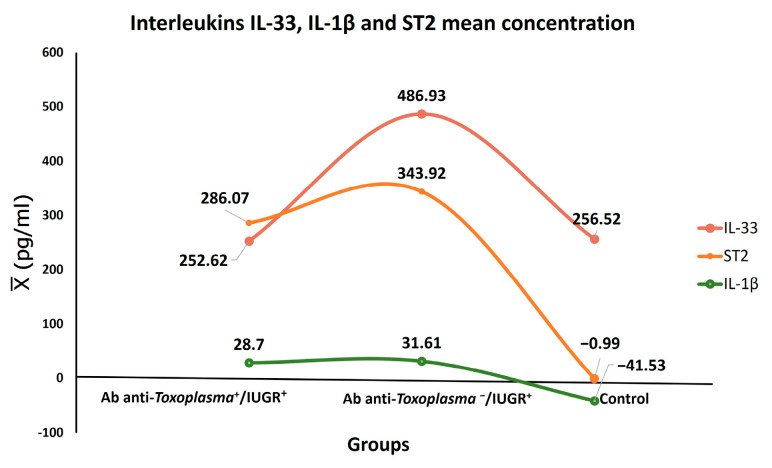
Mean concentrations interleukins by group, Anti-*T. gondii* antibodies+/IUGR+, Anti-*T. gondii*-/IUGR+ and control group.

The highest values for IL-33 and ST2 were found in the Anti-*Toxoplasma* antibodies−/IUGR + group, while the maximum concentration of IL-1β corresponded with the *Toxoplasma* infection+/IUGR+ group (Figure 5).

## 4. Discussion

IL-33 had a close and significant association with IUGR (*p* < 0.001) independently of *T. gondii* infection. Furthermore, the mean concentration of IL-33 was elevated in the toxoplasmosis+/IUGR+ group at 252.62 pg/mL, while it was 486.93 pg/mL in the toxoplasmosis-/IUGR group. On the other hand, IL-33 levels have been recently reported in pregnant women and *T. gondii* infection [16]. These results suggest that IL-33 possibly acts against tissue damage in IUGR. It is worth mentioning that damage to placental tissue is among the various causes of IUGR, according to its pathophysiology [26]. Another possibility is that the IL-33 signaling pathway triggers NF-κB, which regulates maternal cells during pregnancy by suppressing the pro-inflammatory Th1/Th17 profile [27].

IUGR was overall significantly associated with ST2 (*p* < 0.0001), as was IUGR Grade 1 (*p* < 0.006). Thus far, a direct association of ST2 with IUGR has not been reported. However, it has been suggested that the pro-inflammatory cytokines TNF-α and IL-1β are involved in IUGR and preeclampsia [28,29]. Another study has suggested a direct association of ST2 with preeclampsia [29]. On the other hand, studies have been conducted on the association of elevated concentrations of sST2 in women with a fetus of small gestational age (SGA) who have experienced preterm birth and with preeclampsia compared with those who had normal pregnancies. The sST2 concentrations were higher in women with preeclampsia and in those with a fetus of small gestational age (SGA) compared to normal pregnant women [29].

The association of ST2 with abortion did not obtain statistical significance. This result could be due to the low number of samples with referred abortions at 14/84 (17.9%) of cases. However, the association of abortion with high serum levels of ST2 has been reported [15,30].

Another possibility could be the low number of patients who presented abortion in this study (14/84). However, there have been reports of an association of IL-33 with recurrent miscarriages [15], as well as a study conducted in a group of pregnant women with depression showing elevated levels of IL-33 [31].

The prevalence of IgG Anti-*T. gondii* antibodies was low (17.8%) and not associated with IUGR, but opposite results in pregnant women and fetal growth restriction associated with the presence of IgG anti-*T. gondii* antibodies have been reported [25]. However, our result is similar to those of investigations that have shown no association between *T. gondii* antibodies and IUGR due to a low value of positivity for IgM [32,33].

The low prevalence of anti-*T. gondii* antibodies in this study was unexpected. Previous studies have demonstrated a high prevalence in women with high-risk pregnancies in the state of Jalisco [34], as well as in a study from Brazil on pregnant women with a 44% chance of seropositivity [16]. This may be due to better prevention measures related to parasite transmission and the general care that the population had during the COVID-19 pandemic. Finally, the sample size may also have influenced this result.

IL-1β was not significantly associated with the presence of IgG anti-*T gondii* antibodies. This result is the opposite to a study in which IUGR was associated with levels of IL-1β [18]. On the other hand, the presence of IgG antibodies was not significantly associated with IL1-β, IL33, or ST2. On the contrary, significant associations of IgG anti-*T. gondii* antibodies and IUGR have been reported [15,16].

The association of IL-1β with IUGR was not statistically significant due to the number of IL-1β positive samples with IUGR at 6/84 (7.14%) of cases. Another study has reported that the positivity of IL-1β does not necessarily correspond with infection but with the change toward the pro-inflammatory profile that favors term delivery [35]. We did not analyzed this since our samples corresponded with 26 weeks of gestation or higher in a cross-sectional study.

Finally, a hypothesis about IUGR regarding fetal genetic and epigenetic marks in response to a large variety of “stressor” exposures during pregnancy exists according to the Developmental Origin of Health and Disease [36,37]. On the other hand, the cell cycle of *Toxoplasma* and the interconversion between tachyzoites and bradyzoites is key for the pathogenesis of toxoplasmosis, which is epigenetically regulated [37]. The above has given rise to new research on IUGR and *T. gondii* infection.

## 5. Conclusions

IUGR was significantly associated with IL-33 and ST2 positivity based on overall IUGR grade.

There was no association between IL-1β and IUGR.

More research is needed to strengthen the utility of IL-33 and ST2 as biomarkers of IUGR.

## Data Availability

The original contributions presented in the study are included in the article, further inquiries can be directed to the corresponding author.
